# Predictors for mortality due to acute exacerbation of COPD in primary care: protocol for the derivation of a clinical prediction rule

**DOI:** 10.1038/npjpcrm.2016.70

**Published:** 2016-10-20

**Authors:** César Alameda, Ángel Carlos Matía, Verónica Casado

**Affiliations:** 1 Department of Healthcare Resources, Castile-Leon Regional Health Authority, Valladolid, Spain; 2 Los Comuneros Health Centre, Burgos Primary Care Authority, Burgos, Spain; 3 Parquesol Health Centre, West Valladolid Primary Care Authority, Valladolid, Spain; 4 Department of Medicine, Dermatology and Toxicology, University of Valladolid, Valladolid, Spain

## Background

Chronic obstructive pulmonary disease (COPD) is the fourth most common cause of death worldwide.^1^ It is expected that this situation will continue to worsen in the coming decades, mainly due to increased tobacco consumption in low- and middle-income countries.^2^ In countries such as Spain, where there has been a decline in smoking, there is already a notable decline in premature mortality and hospitalisations associated with COPD.^3,4^ Throughout the life of an individual living with COPD, frequent exacerbations impair lung function and quality of life, as well as worsen prognosis and raise the associated costs.^5–14^

One area requiring further research in the 'Research Agenda for General Practice / Family Medicine and Primary Health Care in Europe' is the predictive value of history taking and simple clinical examination.^15^ Although some severity score systems have been published, they have been developed in hospital settings and most contain variables that cannot be collected in the context of primary care.^16–20^ We consider it a top priority to define the signs and symptoms that may better predict the severity of an acute exacerbation of COPD (AECOPD), so that we can deliver optimal diagnostic and therapeutic services to our patients.

## Aims

To derive a clinical prediction rule (CPR) for short-term death following an acute exacerbation of COPD (AECOPD).

## Methods

### Study design

A prospective cohort study in primary care (PC).

### Setting

All health centres (HC) of the Spanish provinces of Burgos, Salamanca, Soria, Valladolid and Zamora, totalling 736,183 inhabitants aged between 40 and 79 years.^21^

### Inclusion criteria

All persons ⩾40 years (to exclude patients with potential asthma) attending a HC of the provinces included in the study between December 2013 and December 2014 who were diagnosed with AECOPD (ICD-9-CM code 491.21) will be included.

### Exclusion criteria

Those without a postbronchodilator ratio between forced expiratory volume in one second and forced vital capacity (FEV_1_/FVC) <0.7 in a previous spirometry and those treated for another exacerbation in the past 4 weeks will be excluded. Such cases are classified as 'treatment failure' or 'relapse' and, therefore, are considered a part of the same episode of AECOPD.^22^

### Outcome criteria

Details of the outcome criteria are shown in Table 1.

### Sample size estimation

In studies of predictive models, it is customary to include 10 events for each predictor variable in addition to the dependent variable. On the basis of epidemiological studies, the probability of death by AECOPD is 1.2% in our setting. Therefore, to study 16 possible predictor variables with the prevalence of events set out above, and assuming a 15% loss, it will be necessary to include 16,292 instances of AECOPD.

### Data extraction

All doctors working in the provinces defined by the study will receive a letter describing the study and requesting their cooperation through an improvement in the collection of clinical data. The data extraction will be carried out through a query to MedoraCyL, the electronic health record of Castile-Leon. The diagnoses made by general practitioners (GPs) will not be reviewed; AECOPD is a clinical diagnosis, and this is a real-world research study. Our objective is to determine the prognosis of what the GPs diagnose as AECOPD with the available resources at the moment of patient care. There will be conditions erroneously diagnosed as AECOPD in the same way that there will be AECOPD erroneously diagnosed as other conditions.

To study the prognosis of the entire AECOPD episode and not the prognosis of each visit to the GP that these patients may perform during the same episode, we will consider visits made 4 weeks after a visit for AECOPD as part of the same episode of AECOPD.^22^ In patients showing multiple values for the same determination during their episode of AECOPD, we will select the one in which the doctor determined that the patient showed the worst general condition. In patients who visit the HC several times, death will be determined within 30 days of the last visit.

### Methods of data analysis and synthesis

We will develop a frequency table and a univariate analysis of the predictive ability of each of the predictor variables, both for the primary and for the secondary end points. The statistical significance of the effect of qualitative variables will be studied with the chi-square test. If the expected frequency is >5 in more than 20% of the instances, the Yates correction will be applied. Regarding quantitative variables, normality will be studied with the Kolmogorov–Smirnov test, and statistical significance of the effect will be studied with Student’s *t*-test.

We will develop a logistic regression for all-cause mortality at 30 days. We will start from the top model, and variables will be phased out following the method of 'successive steps backward.' This process has been developed taking into account the following features:

- Missing values of a variable will be replaced by the average.
- Non-dichotomous categorical variables will be transformed into dummy variables, which may be combined depending on the frequency distribution.
- Continuous variables will be included in the model after checking their linearity. If not present, the necessary changes will be made.
- We will use the likelihood ratio test to remove predictors from our model. The significance value for this test will be 0.1.
- The estimation of the regression coefficients will be performed with the maximum likelihood method.
- The goodness of fit will be studied using the log-likelihood function.

After building the model, we will test its fit with the Hosmer–Lemeshow test and determine its accuracy with the area under the ROC (receiver operating characteristic) curve.

We will build the CPR with this model. The CPR will be presented as a score chart on which a score is assigned to each predictor according to its regression coefficient, to facilitate its clinical use. We will determine two cut-off points for the result of the sum of the scores, and three risk categories for AECOPD mortality will be designated as low, medium and high.

These same steps will be repeated to develop a prognostic model for the secondary end point (death by AECOPD or related complications within 30 days of visit). We will compare the prognostic ability between this model and that for all-cause mortality. If no significant differences are found, the model derived for all-cause mortality at 30 days will also be proposed to predict the secondary end point.

The statistical analyses will be performed using IBM SPSS Statistics 21 for Windows (IBM, Chicago, IL, USA).

### Ethical approval

The study protocol has been approved by the local Research Ethics Committees.

## Short discussion

A significant amount is known regarding the predictors of mortality in people with COPD. However, although most people with COPD die from an exacerbation of this condition, there is little published evidence about predictors of mortality by AECOPD, and all such data are based on results from hospital settings. The main strength of this study is that it will be performed in the context of real-world PC and with a large sample size, so that the applicability of the results to clinical practice may be greater than that of other studies. The main limitation of this study is the low rate of the primary end point, which requires a study of only a few predictors to avoid the risk of overfitting; this may make it more difficult to adjust for confounding factors such as comorbidities. Moreover, the predictors of death by AECOPD found in previous studies showed low sensitivity, which could threaten the discrimination of the CPR. The use of real-world data leads to more lost data, especially with regard to spirometry or vital signs that are not systematically measured in PC, such as respiratory rate. The observational design is a limitation itself because it increases the probability of biases. To generalise the findings of this study and to promote its use, validation and clinical impact studies should also be conducted. With this knowledge, clinicians may recognise the signs of poor prognosis in persons with AECOPD and treat them at an earlier stage and avoid diagnostic tests, treatments and hospitalisations in patients who do not require them. The goal of such studies is the optimisation of resources used for the diagnosis and treatment of people with AECOPD.

## Figures and Tables

**Table 1:** Outcome criteria

*Outcomes*	*Characteristics*
*Primary outcome*	
Death by any cause within 30 days of visit	
*Secondary outcome*	
Death by AECOPD or related complications within 30 days of visit	
*Predictors*	
Sex	
Age	
Respiratory rate	
Peripheral oxygen saturation	
Systolic blood pressure	
Diastolic blood pressure	
Peripheral temperature	
Body mass index	In the last year
Oedema in the legs	
Use of accessory muscles of respiration	
Confusion	Drowsiness, stupor, coma or disorientation to person, place or time
Dyspnoea	According to the mMRC scale
Observed FEV_1_/Predicted FEV_1_	In the last two years
Charlson comorbidity index	
Included in the home care programme.^a^	
Exacerbations treated in last 12 months	Recorded on EHR

Abbreviations: AECOPD, acute exacerbation of chronic obstructive pulmonary disease; EHR, electronic health record; FEV_1_, forced expiratory volume in one second; mMRC, modified Medical Research Council.

a Includes people who spend most of their time in bed (who can only leave with the help of others) and those with significant mobility impairments (preventing them from leaving home, except in exceptional cases), regardless of the illness. The expected duration of this disability is more than 2 months.
